# Ringhalexin from *Hemachatus haemachatus*: A novel inhibitor of extrinsic tenase complex

**DOI:** 10.1038/srep25935

**Published:** 2016-05-13

**Authors:** Bhaskar Barnwal, Chacko Jobichen, Vallerinteavide Mavelli Girish, Chun Shin Foo, J. Sivaraman, R. Manjunatha Kini

**Affiliations:** 1 Department of Biological Sciences, Faculty of Science, National University of Singapore, Singapore 119260, Singapore

## Abstract

Anticoagulant therapy is used for the prevention and treatment of thromboembolic disorders. Blood coagulation is initiated by the interaction of factor VIIa (FVIIa) with membrane-bound tissue factor (TF) to form the extrinsic tenase complex which activates FX to FXa. Thus, it is an important target for the development of novel anticoagulants. Here, we report the isolation and characterization of a novel anticoagulant ringhalexin from the venom of *Hemachatus haemachatus* (African Ringhals Cobra). Amino acid sequence of the protein indicates that it belongs to the three-finger toxin family and exhibits 94% identity to an uncharacterized Neurotoxin-like protein NTL2 from *Naja atra*. Ringhalexin inhibited FX activation by extrinsic tenase complex with an IC_50_ of 123.8 ± 9.54 nM. It is a mixed-type inhibitor with the kinetic constants, Ki and Ki’ of 84.25 ± 3.53 nM and 152.5 ± 11.32 nM, respectively. Ringhalexin also exhibits a weak, irreversible neurotoxicity on chick biventer cervicis muscle preparations. Subsequently, the three-dimensional structure of ringhalexin was determined at 2.95 Å resolution. This study for the first time reports the structure of an anticoagulant three-finger toxin. Thus, ringhalexin is a potent inhibitor of the FX activation by extrinsic tenase complex and a weak, irreversible neurotoxin.

Snake venoms constitute a pharmacological repertoire of various proteins and polypeptides. Venom usually contains hundred or more different proteins that belong to various structural super-families like three-finger toxins (3FTxs), C-type lectin like proteins, phospholipase A_2_s, serine proteases and metalloproteases^1^,^2^,^3^. 3FTxs are a well-characterized family of non-enzymatic polypeptides containing 60 to 74 amino acid residues^4^,^5^. These proteins are abundant in elapid (cobras, kraits and mambas), hydrophiid (sea snakes) and colubrid venoms^6^ and have also been identified in viper venoms^7^,^8^. They contain four to five disulfide bridges, four of which are strictly conserved. They show an analogous pattern of folding, in which three β-stranded loops extend from a central core containing the four conserved disulfide bridges resembling the three outstretched fingers of a hand^4^,^5^. Due to this appearance, this family of proteins is named as 3FTxs. Despite their structural similarity, 3FTxs display a wide range of functional diversity^2^,^5^. They can be broadly classified into neurotoxins^9^, cardiotoxins/cytotoxins^10^ and anticoagulants^11^ based on their mechanism of action.

Snake venom neurotoxins target the neuromuscular junctions in the peripheral and central nervous system thereby interfering with cholinergic transmissions^12^. They can be categorized into α-neurotoxins, κ-toxins and muscarinic toxins that target muscle nicotinic acetylcholine receptors (nAChR), neuronal nAChR and various subtypes of muscarinic receptors, respectively. The short-chain and long-chain α-neurotoxins bind to muscle αβγδ nAChR with equipotency. However, only long-chain but not short-chain neurotoxin bind to neuronal α7 receptor with high affinity^13^,^14^. κ-Toxins bind specifically to neuronal (α3β4) nAChR whereas muscarinic toxins specifically and selectively targets various subsets of muscarinic acetylcholine receptors^15^,^16^. Cardiotoxins/cytotoxins though structurally resemble to short-chain neurotoxins exhibit cardiotoxic and cytolytic effects by targeting phospholipid membranes^10^,^17^,^18^ whereas anticoagulant 3FTxs inhibits a specific protease or coagulation complex in the coagulation cascade.

We are specifically interested to study anticoagulant 3FTxs because of the paucity of knowledge on their structure-function relationships. The anticoagulant and antiplatelet effects of 3FTxs was first described from the venom of *Naja nigricollis crawshawii*^19^. However, the mechanisms of anticoagulant activity of 3FTx were unknown. Recently, we have characterized a 3FTx, naniproin from *N. nigricollis crawshawii* venom, which specifically inhibits the prothrombinase complex (CY Koh, RM Kini, unpublished observations). We also determined the mechanism of action of a novel anticoagulant protein complex, hemextin from the venom of Ringhals cobra (*Hemachatus haemachatus*). The tetrameric hemextin AB complex non-competitively inhibits factor VIIa (FVIIa) with nanomolar affinity^11^. On the other hand exactin isolated from the same venom inhibited the activation of factor X (FX) specifically by extrinsic tenase complex. Interestingly, exactin showed structural similarity to short-chain neurotoxins and exhibited a weak neurotoxicity (VM Girish, RM Kini, unpublished observations).

Here we report the identification, purification and characterization of a novel anticoagulant ringhalexin (Ringhals extrinsic tenase complex inhibitor) from the venom of *H. haemachatus*. Ringhalexin exhibited a mixed-type inhibition to FX activation by the extrinsic tenase complex and also exhibited a weak, irreversible, neurotoxicity on chick biventer cervicis muscle (CBCM) preparations. Further we determined the three-dimensional structure of ringhalexin which revealed that it has a 3FTx fold maintained by four highly conserved disulfide bonds.

## Results

### Purification of the anticoagulant protein ringhalexin

**Figure 1:**
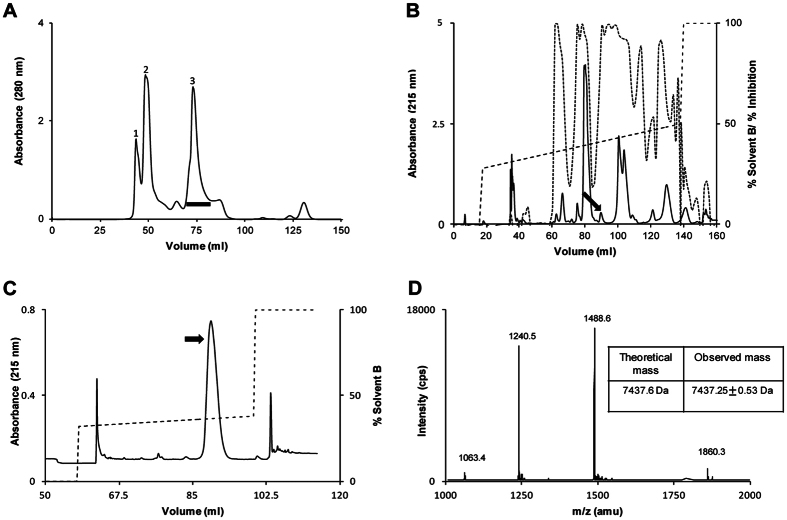
Purification of ringhalexin. (**A**) The crude venom of *H. haemachatus* was sub-fractionated by size-exclusion chromatography (SEC) and the proteins were eluted using 50 mM Tris-HCl (pH 7.4). Peak 3 (horizontal bar) corresponds to non-enzymatic 3FTxs. (**B**) The peak 3 of SEC was subjected to RP-HPLC on a Jupiter C_18_ column (10 × 250 mm). A linear gradient of 28–50% of solvent B was used for the elution of proteins. The inhibitory activities of the individual fractions on FX activation by extrinsic tenase complex were determined (dotted line). The peak indicated by the black arrow contains ringhalexin. (**C**) The fractions containing ringhalexin were pooled and re-chromatographed using a shallow gradient of 32–38% on a Jupiter C_18_ column (4.6 × 250 mm). The peak containing pure ringhalexin is indicated by the arrow. (**D**) ESI-MS of ringhalexin showing four peaks of mass/charge (*m/z*) ratio ranging from +4 to +7 charges. The mass was determined to be 7437.25 ± 0.53 Da.

*H. haemachatus* venom was size-fractionated by Superdex 30 column (Fig. 1A). Three major peaks were obtained and the proteins eluted in peak 3 contained mostly 3FTxs. With the interest of isolating the anticoagulant proteins from 3FTx family, peak 3 was further fractionated on a C_18_ RP-HPLC column. Individual fractions were lyophilized and their inhibitory activities on FX activation by the extrinsic tenase complex were examined. The estimated percent inhibition of each fraction and elution profile (Fig. 1B) indicates the presence of several extrinsic tenase complex inhibitors. Many peaks contained a mixture of different proteins and further purification by various chromatographic techniques resulted in very low yield or showed no inhibition which made further characterization difficult. In this study, we focused on the purification of ringhalexin (*black arrow*) as it was found to be a potent inhibitor of FX activation by the extrinsic tenase complex. Ringhalexin was further purified using a shallow gradient (Fig. 1C). The molecular mass and homogeneity of the purified ringhalexin was determined by electrospray ionization mass spectrometry (ESI-MS). ESI-MS showed 4 peaks of mass/charge (*m/z*) ratio ranging from +4 to +7 charges (Fig. 1D). The mass was determined to be 7437.25 ± 0.53 Da. The total yield of ringhalexin was 1–1.5 mg/g of venom.

### Amino acid sequence of ringhalexin

**Figure 2:**
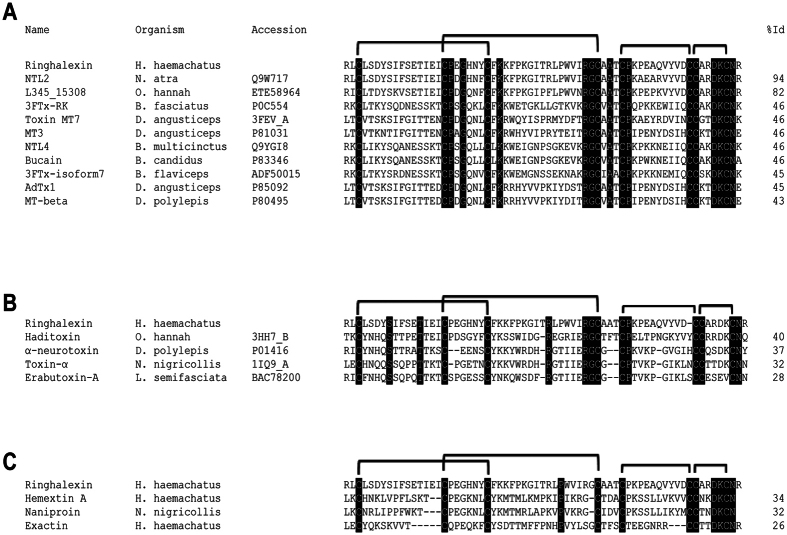
Multiple sequence alignment of novel proteins. Sequence alignment of ringhalexin with the (**A**) most homologous 3FTxs, (**B**) short-chain α-neurotoxins and (**C**) anticoagulant 3FTxs. Toxin names, species, and accession numbers are shown. Conserved residues in all sequences are highlighted in black. The sequence identities (in percentage) of each protein when compared with ringhalexin are shown at the end of each sequence.

The complete amino acid sequence of the ringhalexin was determined by Edman degradation. The first 48 residues were determined by direct sequencing of the native protein whereas the remaining residues were determined by sequencing the overlapping C-terminal fragment of *o*-iodosobenzoic acid cleaved *S*-pyridylethylated ringhalexin (Fig. S1). Sequence alignment and the position of cysteine residues showed that ringhalexin belongs to the 3FTx family. It showed 94% identity to Neurotoxin-like protein NTL2 isolated from *Naja atra* venom (Fig. 2A). However, NTL2 has not been structurally or functionally characterized. It also showed significant identity (82%) to a hypothetical protein L345_15308 of *Ophiophagus hannah* (king cobra). Interestingly, ringhalexin showed low identity to classical short-chain neurotoxins (Fig. 2B) and cytotoxins/cardiotoxins with anticoagulant properties (Fig. 2C).

### β-sheet structure of ringhalexin

The secondary structure of ringhalexin was evaluated by far-UV CD spectroscopy (Fig. S2). The CD spectrum shows a minimum at 217 nm and a maximum at 196 nm. The CD spectrum is comparable to that of haditoxin from *O. hannah* venom with a minimum at 215 nm and maximum at 198–200 nm^20^. However, it differed significantly from that of β-cardiotoxin, a β-blocker from the same venom^21^. Thus, ringhalexin was found to be composed of β-sheet structure similar to all other 3FTxs^4^.

### Ringhalexin inhibits extrinsic tenase complex

**Figure 3:**
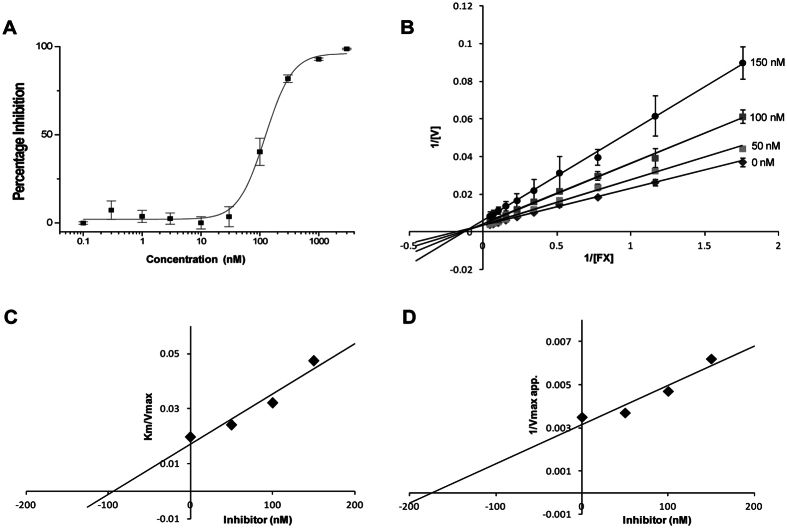
Effect of ringhalexin on reconstituted extrinsic tenase complex. (**A**) Dose-inhibition response of ringhalexin. (**B–D**) Kinetics of inhibition of extrinsic tenase complex. (**B**) The Lineweaver-Burk plot for inhibition of the extrinsic tenase complex by ringhalexin. Decrease in V_max_ and increase in K_m_ with the increase in inhibitor concentration is the characteristic of mixed-type inhibition. Corresponding secondary plots depicting K_i_ (**C**) and K_i_’ (**D**) shows that the affinity of ringhalexin towards the [E] complex (FVIIa/TF_PCPS_) (K_i_) was nearly two times higher than that towards the [ES] complex (FVIIa/TF_PCPS_/FX) (K_i_’). Each data point is the mean ± S.D. of three independent experiments.

We determined the effect of ringhalexin on various clotting times. Ringhalexin significantly prolonged the prothrombin time in a dose-dependent manner. It also prolonged APTT and Stypven time slightly at higher concentration but had no effect on thrombin time (Fig. S3). To determine the potency of ringhalexin, we studied its effect on the reconstituted extrinsic tenase complex. Ringhalexin inhibited FX activation by extrinsic tenase with an IC_50_ of 123.8 ± 9.54 nM (Fig. 3A). However, it does not inhibit FVIIa or FXa amidolytic activity at 10 μM (data not shown). To further understand the interactions, we examined the inhibition kinetics of ringhalexin. The ringhalexin protein showed decrease in V_max_ and increase in K_m_ with the increase in its concentration which is a characteristic of mixed-type inhibition. Thus, ringhalexin exhibits mixed-type inhibition of FX activation by extrinsic tenase complex (Fig. 3B). The kinetic constants, Ki and Ki’ derived from the secondary plot were determined to be 84.25 ± 3.53 nM and 152.5 ± 11.32 nM for FX activation by extrinsic tenase complex (Fig. 3C,D) indicating that the affinity of ringhalexin towards the [E] complex (FVIIa/TF_PCPS_) was nearly two times higher than that towards the [ES] complex (FVIIa/TF_PCPS_/FX).

### Neurotoxic activity of ringhalexin

To observe the biological effects of ringhalexin, the mice were injected with 10 and 100 mg/kg of the protein. No effect was seen at 10 mg/kg dose. At 100 mg/kg, the mice showed typical symptoms of peripheral neurotoxicity such as hind limb paralysis and labored breathing^22^,^23^. The average time of death was recorded to be 96 min. Postmortem examinations showed no internal bleeding or hemorrhage.

**Figure 4:**
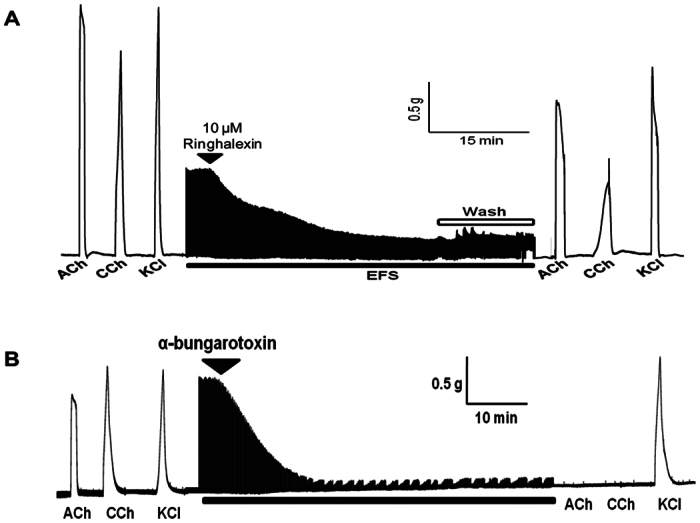
Irreversible neuromuscular blockade produced by ringhalexin in the avian neuromuscular junction. (**A**) The representative segment of tracing showing the blockade of nerve-evoked twitches of the CBCM produced by ringhalexin (10 μM). Upon maximal inhibition of the nerve-evoked twitch responses, the muscle was subjected to extensive washing to remove the toxin from the bath chamber. The muscle was washed with fresh Krebs buffer for 30s carried out at regular intervals of 1 min for 30 min. The black bar indicates EFS (0.2 Hz, 0.1 ms and 7 V) and the hollow bar indicates muscle washing. The recovery is calculated as a percentage of the control twitch responses. (**B**) The representative segment of organ bath tracing showing neuromuscular blockade in isolated CBCM produced by α-bungarotoxin, a postsynaptic neurotoxin isolated from the venom of *Bungarus multicinctus*. Three independent experiments were done with a representative segment shown here.

The effect of purified ringhalexin toxin (1–10 μM) on neuromuscular transmission was studied in the CBCM. Ringhalexin toxin produced time- and concentration-dependent blockade of nerve-evoked twitch responses in indirectly stimulated CBCM. At 10 μM, ringhalexin toxin produced 75% inhibition of the nerve-evoked twitch responses in the avian neuromuscular junction after 30 min exposure to the toxin (Fig. 4A). The KCl-induced contraction was unaffected, indicating the absence of myotoxicity produced by the toxin. The reversibility of the neuromuscular blockade produced by ringhalexin toxin was evaluated through intermittent washing of the muscle with fresh Krebs solution. No recovery of the neuromuscular blockade was observed following washing of the muscle for 30 min, hence the results indicate that the neuromuscular blockade produced by ringhalexin was irreversible. We used α-bungarotoxin, a well-characterized long-chain neurotoxin, as a positive control (Fig. 4B).

### Structural analysis

**Figure 5:**
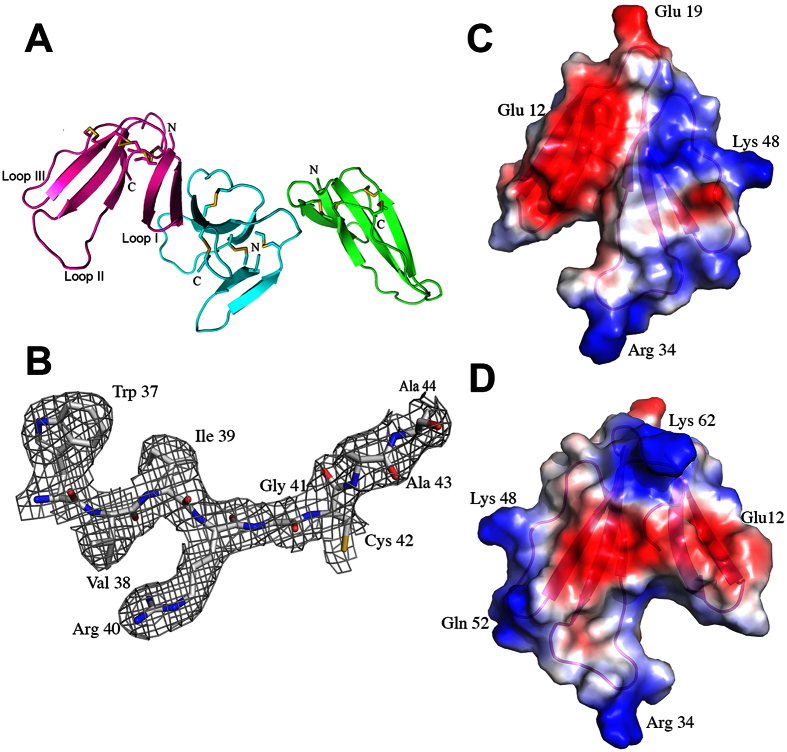
Structure of ringhalexin. (**A**) Cartoon representation of the ringhalexin asymmetric unit. Monomer A: Magenta, Monomer B: Cyan and Monomer C: Green. Cysteine bonds are shown in yellow. N- and C- terminals are labelled. (**B**) Electron density map. A sample final 2Fo-Fc map of ringhalexin shows the region from Trp37 to Ala44. The map is contoured at a level of 1σ. (**C**) The electrostatic surface potential of ringhalexin is shown in the same orientation as in (**A**). (**D**) The electrostatic surface potential of ringhalexin after 180° rotation. Blue indicates positive potential and red indicates negative potential in units kT/e. All the structure related figures of this paper were prepared using the program PyMol^63^.

**Figure 6:**
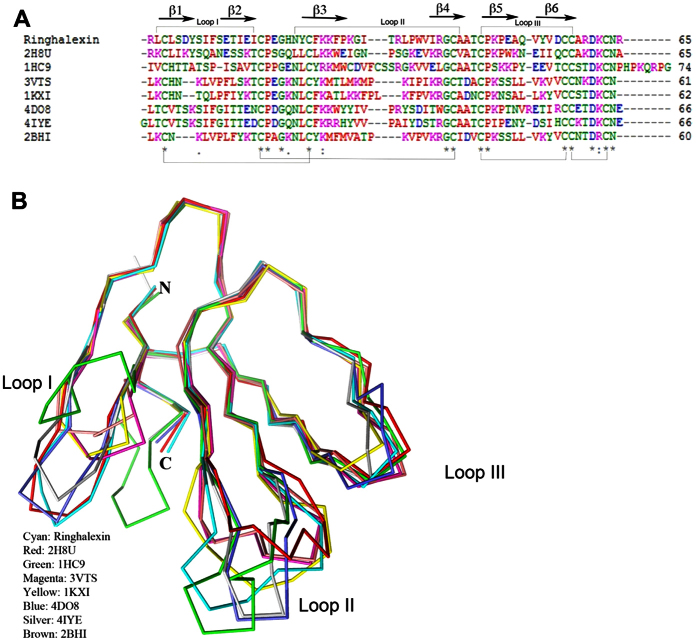
Comparison of ringhalexin with other three-finger toxins. (**A**) Sequence alignment of ringhalexin and its homologs, bucain [2H8U]^64^, α-bungarotoxin [1HC9]^65^, hemachatoxin [3VTS]^66^, cardiotoxin V [1KXI]^67^, muscarinic toxin MT1[4DO8]^68^, ρ-Da1a toxin [4IYE]^69^ and cardiotoxin A3 [2BHI]^70^. This figure was generated using the programs ClustalW^71^. Secondary structural elements of ringhalexin are shown on top and disulfide bridges are shown below. (**B**) Comparison of ringhalexin with its structural homologs. Ringhalexin (Cyan), bucain [2H8U] (red)^64^, α-bungarotoxin [1HC9] (green)^65^, hemachatoxin [3VTS] (magenta)^66^, cardiotoxin V [1KXI] (yellow)^67^, muscarinic toxin MT1[4DO8] (blue)^68^, ρ-Da1a toxin [4IYE] (silver)^69^ and cardiotoxin A3 [2BHI] (brown)^70^.

The structure of ringhalexin was determined by the molecular replacement method using the Balbes program^24^ using *Bungarus candidus* toxin Bucain coordinates (PDB code 2H8U) as model. There were three protein molecules in an asymmetric unit with each molecule consisting of residues from Arg1 to Ala65 (Fig. 5A). All the three monomers are well defined in the electron density map (Fig. 5B). The model was refined to a final R value of 0.22 (R_free_ = 0.27) (Table 1). The stereo-chemical parameters of the model were analysed by PROCHECK and all residues are in the allowed regions of the Ramachandran plot. Each monomer of the asymmetric unit consists of 6 anti-parallel β-strands (β2↓β1↑β4↓β3↑β6↓β5↑) that form two β-sheets (Fig. 5A). The first β-sheet consists of two anti-parallel β-strands, β1 (Leu2-Tyr7) and β2 (Ser11-Ile16), while the second contains four anti-parallel strands, β3 (Tyr23-Pro29), β4 (Ile39-Ala43), β5 (Cys46-Ala51) and β6 (Val53-Cys58). The fold of ringhalexin is maintained by four disulfide bonds, and these cysteines are strictly conserved among the 3FTxs. The three fingers of ringhalexin consist of the secondary structures β1Ωβ2, β3Ωβ4 and β5Ωβ6 (Fig. 5). The electrostatic surface representation shows that the molecule is predominantly positively charged with few negative patches in the surface (Fig. 5C,D). The sequence alignment revealed the conserved residues of ringhalexin as well as its identity to cardiotoxins/cytotoxins (Figs 2 and 6A). Also, ringhalexin shared the common three-finger fold and molecular shape when compared to its structural homologues (Fig. 6B).

A search for topologically similar proteins within the Protein Data Bank (www.pdb.org) with the program DALI^25^ revealed significant structural homology between ringhalexin and other 3FTxs (Table 2). The closest homologs were bucain, cytotoxin and erabutoxin. Interestingly, none of the closest DALI homologs had anticoagulant properties.

### Phylogenetic Analysis

A phylogenetic analysis was performed for ringhalexin to understand the evolutionary relationship among various 3FTxs. Our phylogenetic analysis shows that 3FTxs can be broadly divided into five branches. It can be deduced from the branch lengths of 3FTxs in their respective phylogenetic trees that these sequences have undergone significant evolutionary remodeling (Fig. S4). Ringhalexin appears to be evolutionarily closer to neurotoxin-like protein NTL2 from *Naja atra* and an uncharacterized protein from *Ophiophagus hannah*. Other two 3FTxs which share the same node are muscarinic toxin 38 from *Ophiophagus hannah* and an uncharacterized protein from *Pantherophis guttatus*.

## Discussion

Haemostasis is a subtle, highly regulated system and the precise control of blood coagulation is important for the life of humans as any imbalance in its regulation can lead to excessive bleeding or unwanted clot^26^,^27^,^28^. Coronary heart diseases and cerebrovascular diseases are the major cause of mortality, resulting in most number of deaths than all other causes together in the western world^29^.

Anticoagulants are used for the prevention and treatment of thromboembolic disorders. Although coumarins, such as warfarin, and heparin are widely used anticoagulants, both have their own limitations, such as variable dose response and narrow therapeutic window^30^. Therefore, there is a great need to develop new anticoagulants targeting specific coagulation enzymes or steps in the coagulation cascade^31^. Blood coagulation cascade is initiated by the extrinsic tenase complex which makes it an important target for the novel anticoagulants development^32^,^33^. In the past, several inhibitors directed against extrinsic tenase complex, which is thought to be initiator of the blood coagulation cascade, have been studied as it might achieve a better anticoagulation efficacy^34^. But these inhibitors must be engineered to exert their effects only at the required site without affecting physiological haemostasis. Endogenous protein, tissue factor pathway inhibitor (TFPI), has three Kunitz-type proteinase inhibitor domains. It interacts with FXa via P1 residue (Arg107) in the second Kunitz-type domain followed by inhibiting FVIIa/TF by binding to FVIIa active site. FFR-FVIIa, generated by incorporating a tripeptide in the active site of FVIIa, limits the formation of functional FVIIa/TF complex^35^. In addition, two classes of peptide exosite inhibitors and several synthetic compounds targeting FVIIa active site have been designed but they have major limitations such as non-specific inhibition, insufficient oral bioavailability or incomplete inhibition even at saturating concentrations^36^,^37^,^38^,^39^,^40^,^41^. A soluble TF mutant with alanine substituted for Lys165 and Lys166 (TFAA) was developed as an anticoagulant^42^. Antibodies against TF have been shown to inhibit the proteolytic activation of FX. One type of antibodies interferes with FVIIa/TF association whereas the other type interferes only with macromolecular substrate docking^43^,^44^.

Several natural extrinsic tenase complex inhibitors have also been identified and characterized. Nematode anticoagulant protein c2 (NAPc2), a serine protease inhibitor from canine hookworms, inhibits the catalytic complex of FVIIa/TF by first binding to FXa^45^. In contrast to TFPI, NAPc2 binds at an exosite of FX/FXa. Ixolaris, a two-Kunitz TFPI from Ixodes scapularis, interacts with FX/FXa exosite with its second domain followed by the docking of its first domain into FVIIa/TF active site^46^,^47^. Although various snake venom proteins have been characterized for their anticoagulant properties, the role of 3FTxs as anticoagulants remains to be studied extensively^48^,^49^.

Here we report the isolation, purification and characterization of a novel protein ringhalexin which was identified by activity-based screening of the *H. haemachatus* crude venom. It exhibited low identity to the well characterized short-chain α-neurotoxins and cytotoxins/cardiotoxins with anticoagulant properties (Fig. 2). Ringhalexin inhibits extrinsic tenase complex with an IC_50_ of 123.8 ± 9.54 nM which is comparable to that of hemextin. However, ringhalexin protein shows a mixed-type inhibition in contrast to the non-competitive inhibition exhibited by hemextin^11^. Hemextin AB complex inhibits FVIIa amidolytic and proteolytic activity non-competitively with a Ki of 50 nM. Ringhalexin does not affect the amidolytic activities of FVIIa or FXa.

Kinetic data of exactin, a mixed-type inhibitor of extrinsic tenase complex from the same venom, indicates that its affinity towards [ES] complex (FVIIa/TF_PCPS_/FX) is 5 times higher than that towards [E] complex (FVIIa/TF_PCPS_) (VM Girish, RM Kini, unpublished observations). In contrast, our kinetic data indicates that ringhalexin binds to [E] complex (FVIIa/TF_PCPS_) better than [ES] complex (FVIIa/TF_PCPS_/FX). On the other hand, naniproin from *Naja nigricollis* venom competitively inhibits prothrombin activation by prothrombinase complex. Kinetic assays ascertain that naniproin interferes with FXa-FVa interaction by competing with FVa for FXa binding with a Ki of 1.28 μM (CY Koh, RM Kini, unpublished observations). As expected with their functional studies, ringhalexin shows low sequence identity with exactin, hemextin and naniproin (Fig. 2C).

Ringhalexin showed high sequence identity to an uncharacterized Neurotoxin-like protein NTL2 isolated from *Naja atra*. Upon investigation of neurotoxic effects of ringhalexin, it was found to be irreversible weak neurotoxin. At 10 μM, ringhalexin produced 50% inhibition of the nerve-evoked twitch responses in the avian neuromuscular junction after a 15-min exposure to the toxin. However, the EC_50_ values for short-chain neurotoxin erabutoxin b and long-chain neurotoxin α-bungarotoxin are 80 nM and 25 nM, respectively (data not shown). Thus, ringhalexin is quite less potent in neuromuscular blockage when compared to erabutoxin b and α-bungarotoxin. The functional invariant residues in short-chain and long-chain neurotoxins towards the muscle type receptor (αβγδ) have been identified previously^50^,^51^. The most important residues involved in binding to nicotinic acetylcholine receptors (nAChRs) are Lys27, Trp29, Asp31, Phe32, Arg33, and Lys47. Other residues involved in the recognition are His6, Gln7, Ser8, Ser9, and Gln10 in loop I; and Tyr25, Gly34, Ile36, and Glu-38 in loop II of short-chain neurotoxins^13^. Ringhalexin lacks most of these functional invariant residues explaining its low neurotoxicity.

Ringhalexin, like other 3FTxs, has three β-stranded loops extending from a central core containing four conserved disulfide bonds which resembles the three outstretched fingers of a hand (Fig. 6B). Ringhalexin showed highest structural similarity to bucain, muscarinic toxin and various cardiotoxins (Table 2). The closest structural homolog in the neurotoxin family was Neurotoxin-1 from *Naja naja oxiana* venom. The DALI search did not return any 3FTx with anticoagulant activity from the PDB database and so far this is the first structure of a 3FTx with anticoagulant properties. As shown in the case of other 3FTxs, the loop II of ringhalexin was very flexible and some of the residues showed very high B values. This flexibility suggests the possible role of loop II residues in the function of ringhalexin. Since this is the first report of 3D structure of 3FTx anticoagulant, we are determining the structure of other anticoagulants for structural comparison. In addition, we plan to examine the structure-function relationships of ringhalexin.

In summary, we have structurally and functionally characterized a novel protein named as ringhalexin from *H. haemachatus*. It is quite possible that the protein has different sites for its anticoagulant and neurotoxic activity. This warrants further investigation and in future we would like to understand the detailed mechanism of its action.

## Methods

### Animals

Swiss albino mice were acquired from the National University of Singapore Laboratory Animal Center and acclimatized to the Animal Holding Unit for at least 3 days before the experiments. The animals were kept under standard conditions with food and water available *ad libitum* in a light-controlled room (12 h light/dark cycle, light on 07:00 h) at 23 °C and 60% relative humidity. Domestic chicks were purchased from Chew’s Agricultural Farm, Singapore and delivered on the day of experimentation.

### Purification of the anticoagulant protein ringhalexin

*H. haemachatus* crude venom (South African Venom Suppliers, Louis Trichardt, South Africa) (80 mg) was sub-fractionated by size-exclusion chromatography (SEC) using a Superdex 30 column (1.6 × 60 cm) equilibrated and eluted with 50 mM Tris-HCl buffer (pH 7.4) using an ÄKTA purifier system (GE Healthcare, Uppsala, Sweden). Peak 3 from SEC was further purified by reverse phase–high performance liquid chromatography (RP-HPLC) on a Jupiter C_18_ column (10 × 250 mm) equilibrated with solvent A (0.1% trifluoroacetic acid). The proteins were eluted using a linear gradient of solvent B (80% acetonitrile in 0.1% trifluoroacetic acid). The individual fractions were collected, lyophilized and reconstituted in 100 μl of activation buffer (50 mM HEPES pH 7.4, 140 mM NaCl, 5 mM CaCl_2_, 1% BSA). The inhibitory effects of the reconstituted fractions were examined on FX activation by the extrinsic tenase complex (described below). The peak corresponding to ringhalexin was pooled and re-chromatographed using a shallow gradient of 32–38% on a Jupiter C_18_ column (4.6 × 250 mm). The molecular weight of the protein sample was determined by electrospray ionization-mass spectrometry (ESI-MS) using API-300 LC/MS/MS system (PerkinElmer Life Sciences, Wellesley, MA, USA). Analyst software 1.4.1 was used for the analysis and deconvolution of the raw mass data.

### Determination of amino acid sequence

Ringhalexin (1 mg) was dissolved in 500 μl of denaturation buffer (130 mM Tris-HCl pH 8.5, 1 mM EDTA, 6 M guanidine HCl) which was followed by the addition of the reducing agent β-mercaptoethanol (1.1 μl; 20X molar excess of disulfide bonds). The reaction mixture was incubated under nitrogen gas stream for 3 h at room temperature. The alkylating reagent 4-vinylpyridine (4.7 μl; 3X molar excess of β-mercaptoethanol) was added followed by further incubation of 2 h at room temperature under the nitrogen stream. The *S*-pyridylethylated protein was purified from the reaction mixture on a Jupiter C_18_ column using a linear gradient of 20–60% solvent B. The molecular weight was determined by ESI-MS. For cleavage, *o*-iodosobenzoic acid (1 mg) was dissolved in 100 μl of 80% (v/v) acetic acid containing 4 M guanidine HCl and 2 μl p-cresol. The mixture was incubated for 2 h at room temperature. The purified *S*-pyridylethylated protein (0.4 mg) was added to this chemical mixture and incubated for 24 h at room temperature in the dark. The reaction was terminated by adding 1.9 ml water into the mixture. The cleaved peptides were purified from the reaction mixture on a Jupiter C_18_ column using a linear gradient and the molecular weights were determined by ESI-MS. The N-terminal sequence of native ringhalexin and the cleaved peptides were determined by automated Edman degradation using PerkinElmer Life Sciences Model 494 Pulsed liquid-phase sequencer (Procise, Foster City, CA, USA) with an on-line Model 785A phenylthiohydantoin-derivative analyzer.

### CD spectroscopy

Far-UV CD spectra (260–190 nm) were recorded using a Jasco J-810 spectropolarimeter (Jasco Corporation, Tokyo, Japan). The protein samples (20–50 μM) were dissolved in 1 mM phosphate buffer and the measurements were carried out at room temperature using a 0.1 cm path length cuvette. The instrument optics was flushed with 30 l/min of nitrogen gas. The spectra were recorded using a scan speed of 50 nm/min, resolution of 0.1 nm and bandwidth of 1 nm. An average of three scans was taken to increase the signal to noise ratio and baseline was subtracted.

### Effect of ringhalexin on plasma clotting times

All experimental protocols were approved by Institutional Review Board (NUS-IRB reference code: 08-322E) and the experiments were conducted in accordance with the approved guidelines. Following written informed consent from the healthy volunteers, citrated human blood was obtained through Tissue Repository (National University Hospital, Singapore). Fresh plasma was obtained by centrifugation at 2600 g, 4 °C for 15 min. The effect of ringhalexin (0.3 μM to 100 μM) in 50 mM Tris-HCl buffer, pH 7.4 were studied on Prothrombin time, Stypven time, Thrombin time and APTT of human plasma (described below). All the experiments were done at 37 °C and the fibrin clot formation was monitored using a 96-well microplate reader for 10 min at 650 nm.

### Prothrombin time

Briefly, 100 μl of plasma, 25 μl of 50 mM Tris- HCl, pH 7.4 and 50 μl of ringhalexin were incubated for 5 min which was followed by the addition of 25 μl of pre-warmed thromboplastin with calcium reagent to initiate clotting. The fibrin clot formation was monitored by microplate reader.

### Stypven time

Briefly, 50 μl of plasma was incubated with 50 μl of ringhalexin for 3 min followed by addition of pre-warmed RVV-X (50 μl, 10 ng/ml) and incubated for another 2 min. The clotting was initiated by the addition of 50 μl of 25 mM pre-warmed CaCl_2_ and the fibrin clot formation was monitored.

### Thrombin time

Equal volumes (50 μl) of plasma, 50 mM Tris buffer, pH 7.4 and ringhalexin were incubated together for 5 min. Later, 50 μl of pre-warmed thrombin time reagent (0.15 NIH units) was added and the fibrin clot formation was monitored.

### Activated partial thromboplastin time (APTT)

Equal volumes (50 μl) of plasma and ringhalexin were incubated for 3 min followed by the addition of pre-warmed APTT reagent (50 μl) and incubated further for 2 min. The clotting was initiated by the addition of 50 μl of pre-warmed 25 mM CaCl_2_ and the fibrin formation was monitored.

### Effect of ringhalexin on the activation of FX by the extrinsic tenase complex

The extrinsic tenase complex was reconstituted by incubating 10 pM of human FVIIa (Haemtech, Essex Junction, VT, USA) with 25 μl of reconstituted recombinant human TF (Innovin) (Dade Behring, Marburg, Germany) in an activation buffer (50 mM HEPES pH 7.4, 140 mM NaCl, 5 mM CaCl_2_, 1% BSA) for 15 min at 37 °C which was followed by the addition of various concentrations of ringhalexin (100 pM to 3 μM) and further incubated for 15 min at 37 °C. FX (Haemtech) was added to make a final concentration of 30 nM and incubated for additional 15 min. The reaction was stopped by adding 50 μl of quench buffer (50 mM HEPES pH 7.4, 140 mM NaCl, 50 mM EDTA, 1% BSA) and FXa was measured by the hydrolysis of 500 μM of S-2222 (Chromogenix, Milano, Italy) in a microplate reader at 405 nm. FXa formed in the absence of ringhalexin was considered as 100% and IC_50_ was determined accordingly.

### Kinetics of inhibition

The kinetics of inhibition of FX activation by extrinsic tenase complex (FVIIa/TF_PCPS_) was determined. The reactions were carried out in an assay buffer of 50 mM HEPES pH 7.4, 140 mM NaCl, 5 mM CaCl_2_, 1% BSA at 37 °C and the quench buffer used was 50 mM HEPES pH 7.4, 140 mM NaCl, 50 mM EDTA, 1% BSA. The inhibitory action of ringhalexin was examined over a wide range of substrate concentrations. For FX activation by FVIIa/TF_PCPS_, varying concentration of FX (0.58 nM–50 nM) were added to individual wells of a 96-well plate containing FVIIa (10 pM) in complex with recombinant human TF (Innovin) and ringhalexin (50 nM to 150 nM). After 15 min, FXa generation was quenched and the initial reaction velocities were measured as a linear increase in the absorbance at 405 nm by adding 500 μM S-2222. Data obtained from this study was fitted to the following equation for the mixed-type inhibition to determine the Ki and Ki’ values.


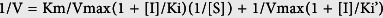


### Neurotoxic activity of ringhalexin

All animal experiments were conducted according to the protocol (021/07a) approved by the Institutional Animal Care and Use Committee of the National University of Singapore.

#### *In vivo* toxicity study

Ringhalexin protein (200 μl in 0.9% saline) was injected intraperitoneally (*i.p.*) into male Swiss albino mice at doses of 10 and 100 mg/kg (n = 2) and the symptoms were observed. The control group was injected with 200 μl of 0.9% saline (n = 2).

#### *Ex vivo* organ bath study

Isolated tissue experiments were conducted as described previously^6^,^52^ using a conventional organ bath (6 ml) containing physiological Krebs-Henseleit buffer of the composition (in mM): 118 NaCl, 4.8 KCl, 1.2 KH_2_PO_4_, 2.5 CaCl_2_, 2.4 MgSO_4_, 25 NaHCO_3_ and 11 D-(+) glucose), pH 7.4. Organ bath chambers were continuously aerated with carbogen (5% carbon dioxide in oxygen) and maintained at 37 °C throughout the experiment. The resting tension of the isolated tissues was maintained between 1–2 g tension and the tissues were allowed to equilibrate for 30–45 min before the start of an experiment. Electrical field stimulation (EFS) was carried out through platinum ring electrodes using a Grass stimulator S88 (Grass instruments, West Warwick, RI, USA). The magnitude of the contractile responses was measured in gram tension. Data were continuously recorded on PowerLab LabChart 6 data acquisition system using a force displacement transducer (Model MLT0201) (ADInstruments, Bella Vista, New South Wales, Australia).

### Chick biventer cervicis muscle (CBCM) preparation

The CBCM nerve-skeletal muscle preparation^53^ was isolated from 3- to 5-day old chicks and mounted in the organ bath chamber under similar experimental conditions as described above. Motor responses of the muscle were evoked by stimulating the motor nerve supramaximally by EFS (7–10 V, 0.1 ms, 0.2 Hz). Submaximal contractures to exogenously applied ACh (200 μM for 30 s), CCh (20 μM for 90 s) and KCl (30 mM for 60 s) were obtained in the absence of EFS prior to the addition of the toxin and after complete blockade of nerve-evoked twitch responses in the muscle. The effect of ringhalexin (1–10 μM; n = 3) or α-Bungarotoxin (0.1 μM; n = 3) on nerve-evoked twitch responses of the CBCM was studied. Neuromuscular blockade was expressed as a percentage of the original twitch height after exposure of the CBCM to the toxin. The recovery of the CBCM from neuromuscular blockade produced by the toxin was assessed by washing out the toxin by bath overflow with fresh Krebs solution until maximal recovery.

### Crystallization and structure determination

Crystallization screens were performed with the hanging drop vapor diffusion method using Hampton Research screens. The protein was at a concentration of 35 mg/ml, and 1:1 crystallization drops were set up with the reservoir solution. The diffraction quality crystals of ringhalexin were obtained from a reservoir solution containing 29% MPD + 0.1 M HEPES pH 7 + 0.3 M sodium citrate. Crystals were grown up to 10 days and were cryo-protected with 20% (w/v) glycerol supplemented (the mother liquor concentration was maintained by exchanging water with glycerol) with the crystallization condition. Ringhalexin crystal diffracted up to 2.95 Å resolution and belongs to P4_1_2_1_2 space group. A complete data set was collected using a Saturn944 CCD detector mounted on Rigaku X-ray generator. The data set was processed and scaled using Mosflm^54^ and Aimless^55^. The structure of ringhalexin was determined by the molecular replacement method using the online program Balbes^24^. Bucain, a cardiotoxin from the Malayan Krait *Bungarus candidus* (PDB code 2H8U; sequence identity 45%) was used as a search model. There were three ringhalexin molecules located in the asymmetric unit. The resultant electron density map was of good quality. Several cycles of model building/refitting using the program Coot^56^, and alternated with refinement using the program Phenix-refine^57^, lead to the convergence of R-values (Table 1). Non-crystallographic symmetry (NCS) restraints were used throughout the refinement process.

### Sequence Alignment and Phylogenetic Analysis

Representative 3FTxs homologs were selected from a BLAST search with ringhalexin sequence and used for phylogenetic analysis and tree building. It was performed using the Phylogeny.fr software platform using the “advanced” mode^58^. The sequence alignment was done using MUSCLE^59^, curation using G-blocks^60^, phylogeny using PhyML^61^, and tree building using TreeDyn^62^. The bootstrapping value in the phylogeny mode was set to 100 iterations.

## Additional Information

**Accession Numbers:** The protein sequence data reported in this paper will appear in the UniProt Knowledge base under the accession number C0HJT5. The three dimensional coordinates and structure factors of ringhalexin were deposited in the RCSB (www.pdb.org) database with the access code 4ZQY.

**How to cite this article**: Barnwal, B. *et al.* Ringhalexin from *Hemachatus haemachatus*: A novel inhibitor of extrinsic tenase complex. *Sci. Rep.* **6**, 25935; doi: 10.1038/srep25935 (2016).

## Supplementary Material

Supplementary Information

## Figures and Tables

**Table 1:** Crystallographic data and refinement statistics.

Data collection*	
Unit Cell (Å)	a = 82.16, b = 82.16, c = 82.61
Resolution range (Å)	15-2.95
Wavelength (Å)	1.5418
Observed reflections	53485
Unique reflections	6274
Completeness (%)	99.0 (99.5)
Redundancy	8.5 (8.8)
R_sym_^a^	0.122 (0.67)
I/SigI	14.7 (3.3)
Space group	P4_1_2_1_2
**Refinement**	
Resolution range (Å) I > σ(I)	15–2.95
R_work_^b^	0.22
R_free_^c^	0.27
Root mean square deviation	
Bond lengths (Å)	0.01
Bond angles (°)	1.599
Average B-factors (Å^2^)	
Protein atoms (1514 atoms)	58
**Ramachandran statistics**	
Most favored and allowed regions (%)	100
Disallowed regions (%)	0

Statistics from the current model.

^a^R_sym_ = Σ|I_i_–<I> |/Σ|I_i_| where I_i_ is the intensity of the i^th^ measurement, and <I> is the mean intensity for that reflection.

^b^R_work_ = Σ| F_obs_–F_calc_|/Σ|F_obs_| where F_calc_ and F_obs_ are the calculated and observed structure factor amplitudes, respectively.

^c^R_free_ = as for R_work_, but for 10.0% of the total reflections chosen at random and omitted from refinement.

^*^Values in the parenthesis are the highest resolution bin values.

**Table 2:** Structural similarity of ringhalexin with 3FTxs.

Chain	Z	rmsd	lali	nres	%id	Description
2h8u-A	12.3	1.9	65	65	46	BUCAIN;
4do8-B	11.5	1.7	65	66	46	MUSCARINIC TOXIN 1;
4iye-A	11.4	2.3	65	66	45	TOXIN ADTX1;
3fev-A	10.6	2.5	65	65	46	FUSION OF MUSCARINIC TOXIN 1, MUSCARINIC M1-TOXIN
3neq-A	10	1.9	61	66	44	MUSCARINIC M1-TOXIN1, MUSCARINIC TOXIN1;
2bhi-B	10	1.9	58	60	34	CYTOTOXIN 3;
1kxi-A	9.9	2.1	61	62	41	CARDIOTOXIN V;
3vts-B	9.8	2.2	60	61	37	CYTOTOXIN 1;
1h0j-B	9.7	1.9	58	60	34	CARDIOTOXIN-3;
1ff4-A	9.6	3.9	65	65	42	MUSCARINIC TOXIN/ACETYLCHOLINE RECEPTOR BINDING
4om5-C	9.6	2	58	60	40	CYTOTOXIN 4;
1hc9-A	9.6	2.9	63	74	35	ALPHA-BUNGAROTOXIN ISOFORM V31;
1f94-A	9.6	2.8	61	63	26	BUCANDIN;
3plc-B	9.6	2.2	59	59	44	BETA-CARDIOTOXIN OH-27;
4uy2-D	9.5	3	63	73	35	NEURONAL ACETYLCHOLINE RECEPTOR SUBUNIT ALPHA-9;
1v6p-B	9.5	1.9	59	62	31	COBROTOXIN;
4om4-B	9.5	2	59	60	37	CYTOTOXIN 2;
1vyc-A	9.4	3.9	65	65	46	BUCAIN;
1cdt-A	9.4	1.9	58	60	36	CARDIOTOXIN VII4;
1ntn-A	9.4	2.4	61	72	34	NEUROTOXIN I;
3ebx-A	9.4	2.3	60	62	28	ERABUTOXIN B;
1qkd-A	9.4	2.1	58	62	29	ERABUTOXIN A;
1ug4-A	9.4	2.3	59	60	34	CYTOTOXIN 6;
2qc1-A	9.4	2.8	62	74	35	ALPHA-BUNGAROTOXIN;
2era-A	9.4	2.2	60	62	28	ERABUTOXIN A;
1kba-A	9.3	2.3	62	66	35	KAPPA-BUNGAROTOXIN;
4lft-A	9.3	2.5	61	64	44	ALPHA-ELAPITOXIN-DPP2A;
1qkd-B	9.3	2.1	58	62	29	ERABUTOXIN A;
4om4-D	9.3	2.3	59	60	37	CYTOTOXIN 2;
3era-A	9.3	2.1	58	62	29	ERABUTOXIN A;
1qke-A	9.3	2.3	61	62	30	ERABUTOXIN A;
3era-B	9.2	2.1	58	62	29	ERABUTOXIN A;
6ebx-A	9.2	2.1	58	62	29	ERABUTOXIN B;
5ebx-A	9.2	2.4	61	62	30	ERABUTOXIN A;
1xt3-A	9.2	1.5	57	60	37	CYTOTOXIN 3;
3hh7-A	9.1	2.5	61	65	41	MUSCARINIC TOXIN-LIKE PROTEIN 3 HOMOLOG
4lft-B	9.1	2.7	61	72	43	ALPHA-ELAPITOXIN-DPP2A;
2vlw-B	9.1	2.6	61	65	44	MUSCARINIC M1-TOXIN1;
1iq9-A	9.1	2.4	58	61	34	ALPHA-NEUROTOXIN;
4hqp-H	9	2.8	62	73	35	ALPHA7 NICOTINIC RECEPTOR CHIMERA;
1onj-A	9	2.1	57	61	35	COBROTOXIN B;
1vb0-A	9	2.1	58	61	34	COBROTOXIN B;
1tgx-B	8.9	2.3	59	60	34	GAMMA-CARDIOTOXIN;
2ccx-A	8.9	1.7	58	60	31	CARDIOTOXIN CTX IIB;
2mj0-A	8.8	2.1	60	66	43	WEAK TRYPTOPHAN-CONTAINING NEUROTOXIN;
1ijc-A	8.8	3.2	61	63	26	BUCANDIN;
4om5-B	8.7	2.5	58	60	40	CYTOTOXIN 4;
1cvo-A	8.7	2	62	62	40	CARDIOTOXIN V;
