# Supplementary material for: Ringhalexin from Hemachatus haemachatus: A novel inhibitor of extrinsic tenase complex
Source: Sci Rep. 2016 May 13;6:25935. doi: 10.1038/srep25935 (PMC4865804; doi:10.1038/srep25935)
Supplement: Supplementary Information [file srep25935-s1.doc]

**Ringhalexin from *Hemachatus haemachatus*: A novel inhibitor of extrinsic tenase complex**

Bhaskar Barnwal, Chacko Jobichen, Vallerinteavide Mavelli Girish, Chun Shin Foo, J. Sivaraman, and R. Manjunatha Kini^*^

Department of Biological Sciences, Faculty of Science, National University of Singapore, Singapore 119260, Singapore

**Figure Legends**

**Figure S1: Amino acid sequence determination of ringhalexin. (A)** The S**-**pyridylethylated ringhalexin (black arrow) was purified on a linear gradient of 20–60% solvent B. **(B)** The ESI-MS profile of S-pyridylethylated ringhalexin showing the four peaks of mass/charge (m/z) ratio ranging from +5 to +8 charges. The mass was determined to be 8287.1 ± 0.83 Da. **(C)** The cleaved peptides were purified from the reaction mixture by using a linear gradient of 0-100% solvent B. The C-terminal peptide is indicated by the solid arrow and N-terminal peptide is indicated by the open arrow. **(D)** The ESI-MS profile of the C-terminal peptide showing the three peaks of mass/charge (m/z) ratio ranging from +3 to +5 charges. The mass was determined to be 3596.7 ± 0.65 Da. **(E)** N-terminal sequence determination of native and C-terminal peptide to complete the amino acid sequence. ‘X’ denotes blank cycles which corresponded to conserved cysteine residues in 3FTxs.

**Figure S2: Far-UV CD spectra for ringhalexin, haditoxin and β-cardiotoxin.** The CD spectrum for ringhalexin showed a minimum at 217 nm and a maximum at 196 nm typical of β–sheeted structure. All three proteins were dissolved in 1 mM phosphate buffer and the measurements were carried out at room temperature using a 0.1 cm path length cuvette.

**Figure S3: Effect of ringhalexin on the coagulation of human plasma.** Ringhalexin significantly prolonged the prothrombin time in a dose-dependent manner. Ringhalexin also prolonged APTT and Stypven time slightly but had no effect on thrombin time. Each data point is the mean ± S.D. of three independent experiments.

**Figure S4: Phylogenetic tree.** It shows the evolutionary distance between rhingalexin and its homologs. The numbers in red indicate the confidence levels obtained from bootstrapping for that particular node. The proteins are indicated by the Uniprot accession numbers. Ringhalexin appears to be evolutionarily closer to neurotoxin-like protein NTL2 from *Naja atra* [Q9W717] and an uncharacterized protein from *Ophiophagus hannah* [V8N9N7]. Other 3FTxs which share the same node are muscarinic toxin 38 from *Ophiophagus hannah* [Q2VBN0] and an uncharacterized protein from *Pantherophis guttatus* [A0A098LYI1].

**Figure S1**

**
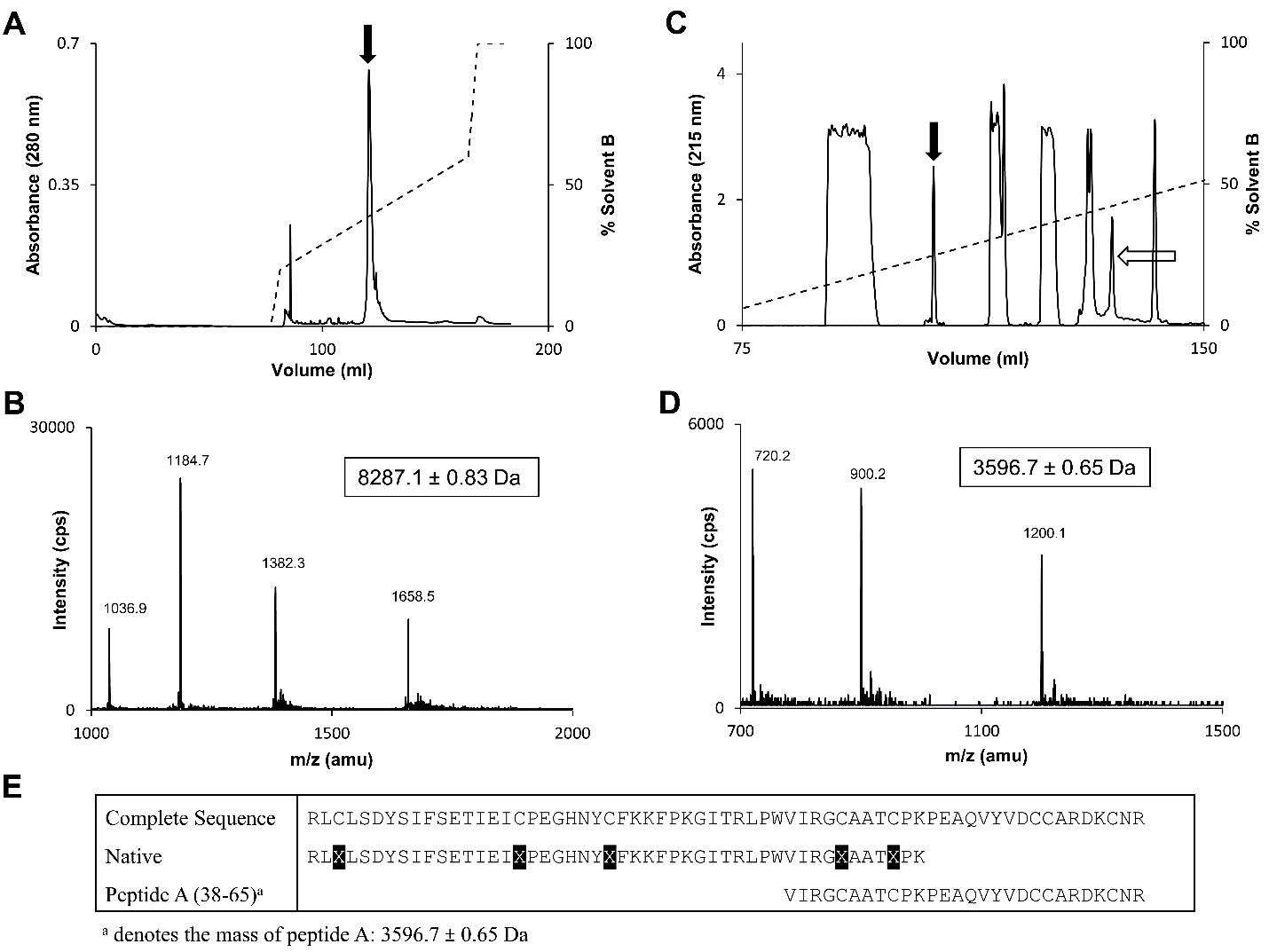
**

**Figure S2**

**Figure S3**

**Figure S4**
